# Identification, Discrimination, and Discovery of Species of Marine Planktonic Ostracods Using DNA Barcodes

**DOI:** 10.1371/journal.pone.0146327

**Published:** 2016-01-05

**Authors:** Lisa M. Nigro, Martin V. Angel, Katarzyna Blachowiak-Samolyk, Russell R. Hopcroft, Ann Bucklin

**Affiliations:** 1 Department of Marine Sciences, University of Connecticut, Groton, Connecticut, United States of America; 2 National Oceanographic Centre, Southampton University, Southampton, United Kingdom; 3 Arctic Ecology Group, Institute of Oceanology, Polish Academy of Sciences, Sopot, Poland; 4 Institute of Marine Science, University of Alaska, Fairbanks, Alaska, United States of America; University of Guelph, CANADA

## Abstract

The Ostracoda (Crustacea; Class Ostracoda) is a diverse, frequently abundant, and ecologically important component of the marine zooplankton assemblage. There are more than 200 described species of marine planktonic ostracods, many of which (especially conspecific species) can be identified only by microscopic examination and dissection of fragile morphological characters. Given the complexity of species identification and increasing lack of expert taxonomists, DNA barcodes (short DNA sequences for species discrimination and identification) are particularly useful and necessary. Results are reported from analysis of 210 specimens of 78 species of marine planktonic ostracods, including two novel species, and 51 species for which barcodes have not been previously published. Specimens were collected during 2006 to 2008 from the Atlantic, Indian, and Southern Oceans, Greenland Sea and Gulf of Alaska. Samples were collected from surface to 5,000 m using various collection devices. DNA sequence variation was analyzed for a 598 base-pair region of the mitochondrial cytochrome oxidase subunit I (COI) gene. Kimura-2-Parameter (K2P) genetic distances within described species (mean = 0.010 ± 0.017 SD) were significantly smaller than between species (0.260 + 0.080), excluding eight taxa hypothesized to comprise cryptic species due to morphological variation (especially different size forms) and/or collection from different geographic regions. These taxa showed similar K2P distance values within (0.014 + 0.026) and between (0.221 ± 0.068) species. All K2P distances > 0.1 resulted from comparisons between identified or cryptic species, with no overlap between intra- and interspecific genetic distances. A Neighbor Joining tree resolved nearly all described species analyzed, with multiple sequences forming monophyletic clusters with high bootstrap values (typically 99%). Based on taxonomically and geographically extensive sampling and analysis (albeit with small sample sizes), the COI barcode region was shown to be a valuable character for discrimination, recognition, identification, and discovery of species of marine planktonic ostracods.

## Introduction

Ostracods (Crustacea, Ostracoda) are a diverse group, which includes over 200 described species occurring in the marine zooplankton assemblage [1]. Planktonic ostracods are opportunistic feeders and primarily eat detritus; they are thought to play an important role in the cycling of organic carbon below the thermocline [2]. They have been documented to be sensitive to water temperature and salinity changes, making them potential indicators of climate change [3,4]. Despite their high abundance in mesozooplankton samples (they are often second only to copepods), the role of ostracods in pelagic communities is largely unknown and almost certainly underestimated. This oversight likely results from subtle morphological characters discriminating species, small size (adult length range from 0.5 to 5 mm), and continuing uncertainties about systematic relationships at the genus level [5]. Notably among the numerous–and frequently systematically complex–taxonomic groups represented in the marine zooplankton assemblage, species of ostracod are extremely difficult to identify based on microscopic morphological characters. Also, even more than for other zooplankton groups, there is a lack of taxonomic expertise for the group: only two or three active researchers currently have sufficient expertise for species identification and description of preserved material, let alone fresh material.

There are numerous morphologically-similar congeneric or sibling species among the halocyprid ostracods that are distinguished mainly by size (i.e., adult carapace length) [6]. Carapace length is a very consistent character within halocyprid species: typical intraspecific size ranges are ±7% of the mean length. A number of sibling species pairs have been discriminated based on this character, including *Orthoconchoecia bispinosa / O*. *secernenda* [7]*; Paramollicia plactolycos* / *P*. *major* [8]; and *Mamilloecia* (*= Paraconchoecia) mamillata* /*M*. *nanomamillata* [9, 10]. Similarly, the most consistent morphological disparity between different populations of halocyprid species is carapace length; populations of differently sized forms are known for a number of species [4, 11]. These different size forms have been generally collected from different geographic locations or depths, but have not previously been analyzed for evidence of genetic differentiation.

Integrative morphological and molecular taxonomic analysis is particularly useful and necessary to develop accurate tools for species identification of ostracods, and thereby to ensure valid estimates of their diversity, distribution, and abundance in routine taxonomic analysis of zooplankton samples. Up-to-date taxonomic information for planktonic ostracods is now widely available through online atlases that provide both taxonomic illustrations and bathymetric profiles of ostracod species for the Atlantic and Southern Oceans [5, 11]. Enhanced attention to the importance of taxonomic training and easier access to taxonomic references materials and keys [12] will also encourage and facilitate improved accuracy and reliability of routine species identification. The use of DNA barcodes (i.e., short DNA sequences useful for species discrimination and identification) [13] for species identification, discrimination, and discovery has also opened new opportunities for integrative morphological–molecular taxonomic analysis of many and diverse groups of marine Metazoa [14]. The DNA sequence most utilized for DNA barcoding of Metazoa is a ~650 base-pair portion of the mitochondrial cytochrome oxidase I gene (COI) defined by consensus–if not quite "universal"–PCR primers [15]. The usefulness of the COI barcode region for species identification results from the fortuitous pattern of variation: intraspecific variation is typically small (< 3% sequence difference), interspecific variation is much larger (15–20%), so there is a marked "barcode gap" between these [16]. Additional attributes are typical of mitochondrial genes: maternal clonal inheritance, high copy number, and a mutation rate that typically results in high sequence variation between even closely-related species [17].

The COI barcode region has proven useful for species identification and discrimination of important groups of marine zooplankton, including comparative analyses of crustacean groups [13, 18, 19, 20, 21, 22]. Additional studies have focused on particular functional groups of marine zooplankton, including copepods [23]; chaetognaths [24]; euphausiids [25]; gastropods [26]; and Medusozoa [27]. Very few studies have included COI barcode data for identified marine planktonic ostracods. Bucklin et al. [28] reported barcodes for 27 species (these data are included in these new analyses); barcode sequences for three additional species have been determined [29, 30].

The widespread use of the COI barcode region sequence has resulted in its use as a new standard for marine biodiversity research and assessment [31]. The COI barcode region has, however, proven problematical for some taxa for a variety of reasons, including lack of resolution between intraspecific and interspecific variation [32] and lack of sequence divergence between species (e.g., for some cnidarians [33].

A common approach to species identification for taxonomically complex groups is toassume that closely-related species occupy distinct biogeographical ranges, but in fact planktonic species may be found in the same sample, due to sample collection across either distributional boundaries and/or through broad bathymetric ranges. Not only is our understanding of biogeographical distributions of zooplankton imperfect, but species ranges may also be changing due to climate, ocean circulation patterns, and other environmental factors [34].

This study presents a comprehensive database of 210 DNA barcodes for 78 identified ostracod species collected from diverse ocean regions, including 51 species for which barcodes have not previously been reported. The described species analyzed show patterns of diversity, differentiation, and divergence within and between species for the COI barcode region that can help ensure routine and accurate identification and discrimination of ostracod species. These data provide a useful resource for integrative molecular and morphological taxonomic analysis of marine planktonic ostracods. Use of DNA barcodes in species identification will allow the inclusion of this abundant, diverse, ecologically important, and geographically widespread group in marine biogeographic and biodiversity research and assessments.

## Materials and Methods

### Sample collection

**Fig 1:**
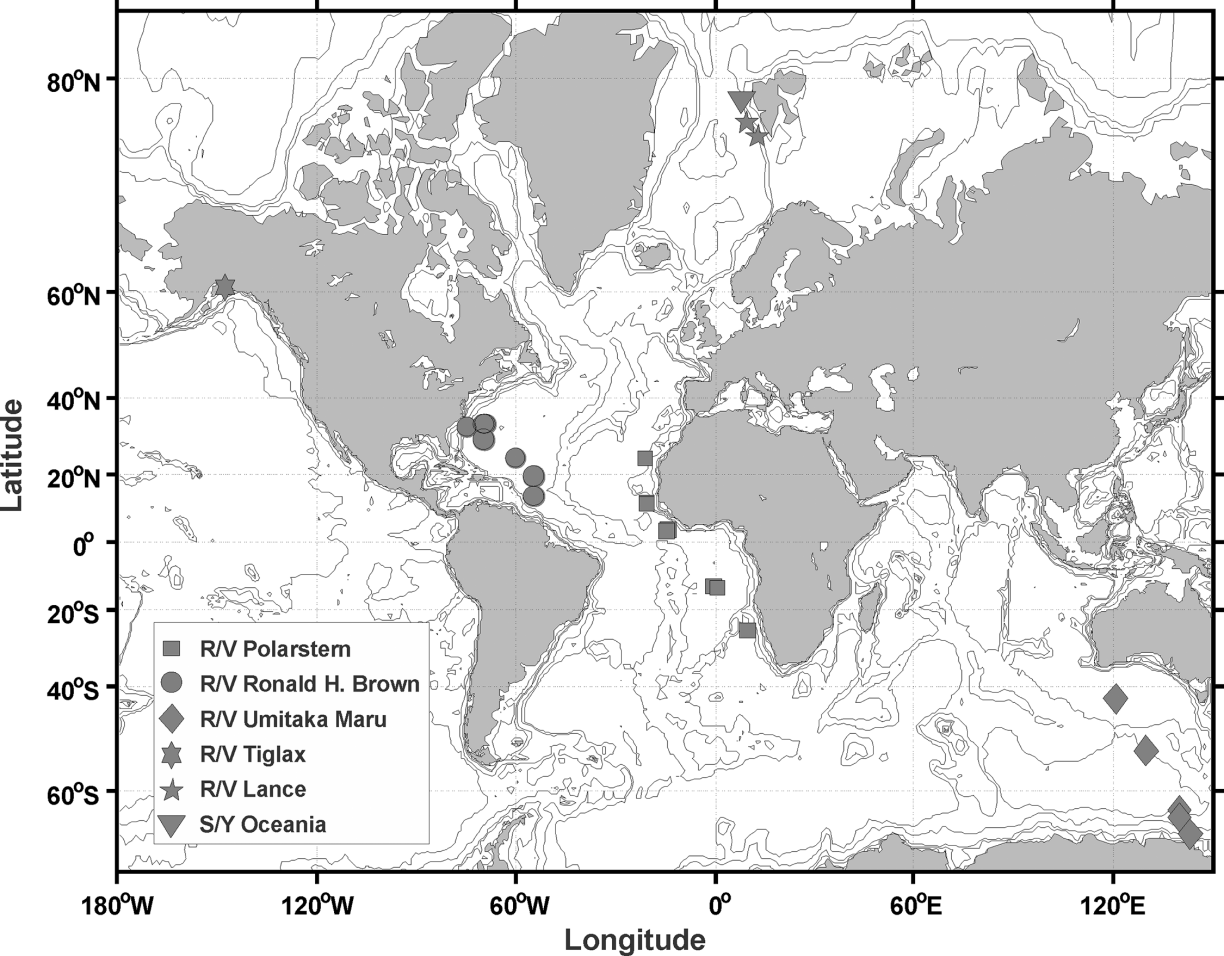
Collection information for samples analyzed in this study. Collection locations of samples from which ostracods were identified for this study. Symbols indicate cruises: R/V *R*.*H*. *Brown* (April, 2006); R/V *Polarstern* (November, 2007); R/V *Umitaka Maru* (January, 2008); R/V *Tiglax* (September, 2008); R/V *Lance* (April, 2008); and S/V *Oceania* (July, 2008). See S1 Table for detailed information of sample collection.

Ostracods were collected across a broad range of ocean regions and depths, from the surface to 5,000 m. The majority of samples were collected from the Atlantic Ocean. Collection methods for two cruises in the Atlantic Ocean, the R/V *R*.*H*. *Brown* (April, 2006), and the R/V *Polarstern* (November, 2007) were described by Wiebe et al. [35]. Additional samples were collected from diverse ocean regions during several different research cruises to: South Indian and Southern Ocean on the R/V *Umitaka Maru* in January, 2008; Gulf of Alaska on the R/V *Tiglax* in September, 2008; and Greenland Sea on two cruises, the R/V *Lance* in April, 2008, and the R/V *Oceania* in July, 2008 (Fig 1). Sampling gear varied among the cruises: a Multiple Opening-Closing Net and Environmental Sensing System (MOCNESS) [36] was used for the R/V *R*.*H*. *Brown* and R/V *Polarstern* cruises; the Multi-net (MN-1, MNS-4), and Rectangular Midwater Trawl (RMT 8+1) systems were used for sampling from the R/V *Umitaka Maru*. Ring nets were used from the R/V *Tiglax*, R/V *Lance*, and S/V *Oceania*. In the latter two expeditions, WP2 nets with 500 μm mesh size were used.

No permits and approvals were required for the oceanographic field sampling for this work, which was carried out in international waters under the auspices of the respective funding agencies. The field studies did not involve any endangered or protected species. Specimens and specimen DNA have been archived at the University Connecticut and all available for examination by researchers with relevant interests and expertise.

### Identification of specimens

Ostracods were identified by microscopic examination of specimens for previously-documented diagnostic morphological characters [5, 11]. During the R/V *Brown* and R/V *Polarstern* cruises, identification was done of living specimens prior to preservation in 95% ethanol [6]; specimens collected during other cruises were identified from ethanol-preserved samples. Identification of specimens collected from the NW Atlantic / Sargasso Sea (R/V *Brown*), Gulf of Alaska *(*R/V *Tiglax)*, and Southern Ocean *(*R/V *Umitaka-Maru)* was done by M.V. Angel; specimens collected from the eastern Atlantic (R/V *Polarstern*) were identified by M.V. Angel and K. Blachowiak-Samolyk; specimens collected from the Arctic (R/V *Lance* and S/Y *Oceania*) were identified by K. Blachowiak-Samolyk.

Among the specimens, at least two novel species were recognized: *Chavturia abyssopelagica* [37] and an undescribed *Bathyconchoecia* species. Specimens that could not be confidently identified to species using traditional morphological characters, usually because of remaining systematic or taxonomic uncertainties at the genus or species level, included some species of *Mikroconchoecia*, *Halocypris*, *Conchoecia*, and *Metaconchoecia*. To aid subsequent analyses, COI barcode sequences were determined and reported for specimens of these genera for which species names could not be confidently assigned (see S1 Table).

### DNA extraction, PCR, and sequencing

Samples for molecular analysis were preserved in 95% undenatured ethanol and identified under a stereomicroscope. Morphological characteristics that varied slightly from the described morphology of the species were carefully noted. For a portion of the samples collected on the R/V *Brown* and R/V *Polarstern*, all molecular methods (including DNA sequencing) were performed at sea. The remaining samples were transported to the University of Connecticut for processing. Voucher specimens have been archived at the University of Connecticut in accordance with the protocols and standards of the Census of Marine Zooplankton (CMarZ; see http://www.cmarz.org/protocols.html). In addition, living images are available for a number of the analyzed species at the CMarZ photo gallery website (see http://www.cmarz.org/.

DNA was extracted from specimens with the DNeasy Blood and Tissue kit (Qiagen, Valencia, California). A ~680 base-pair fragment of the mitochondrial cytochrome oxidase subunit I (COI) gene was amplified either in an Applied Biosystems 9600 Thermal cycler or in a Perkin Elmer 480 thermal cycler using the Gotaq Flexi DNA polymerase (Promega) and the manufacturer’s standard application recommendations for concentrations of buffer, dNTPS, and magnesium chloride. PCR was initially performed with conserved consensus primers [15]: LCO 1490F (5’-GGTCAACAAATCATAAAGATATTGG-3’) and HCO 2198R (5’-TAAACTTCAGGGTGACCAAAAAATCA-3’). A reverse PCR primer specially designed for ostracods, Ost-COI-1535 (5’-GGDGCHTGAAGWGCWATGYTAGG-3’), was used for samples that failed to amplify with HCO 2189R. The PCR conditions were 94°C for 1 min, 45°C for 2 min, and 72°C for 3 min, for 40 cycles in the Perkin Elmer 480 thermal cycler. For the ABI 9600 thermal cycler the conditions were an initial denaturation at 95^o^ C for 3 min; 35 cycles of 95°C for 45 sec; 45°C for 1 min, and 72°C for 1.5 min, and a final extension of 72°C for 3 minutes. PCR products were electrophoresed in 1% agarose and visualized after staining with ethidium bromide. Positive PCR products were purified using Qiagen’s PCR purification kit and sequenced with an Applied Biosystems 3130 capillary DNA Sequencer with 1/8 of the manufacturer’s recommendation of BigDye.

### Data analysis

Sequences were manually edited in Sequencher (Genecodes, Ann Arbor MI) and analyzed for correct amino acid translation. BLAST searches [38] were performed to compare sequences to published nucleotide sequences in the GenBank database. Edited sequences were aligned with MAFFT Ver. 6.7 [39]. Pairwise nucleotide distances were also calculated in MEGA with a K2P model to determine genetic variation within and between ostracod species and genera. Phylogenetic trees were constructed using the Neighbor Joining algorithm in MEGA Ver. 4.1 [40] with a Kimura-2-Parameter (K2P) model [41]. The number of molecular operational taxonomic units (MOTU) [42] was estimated and compared with the number of described species using the software jMOTU [43] for the 598 bp alignment. The minimum alignment length (i.e., overlap between sequence pairs) for analysis was set at 359 bp. The BLAST filter was set at 97%.

## Results and Discussion

Analysis of the COI sequences for 212 specimens of 78 species collected from diverse ocean regions indicates that the barcode region is broadly useful as an additional character for species discrimination, recognition, identification, and discovery.

### Genetic distances within and between species

**Fig 2:**
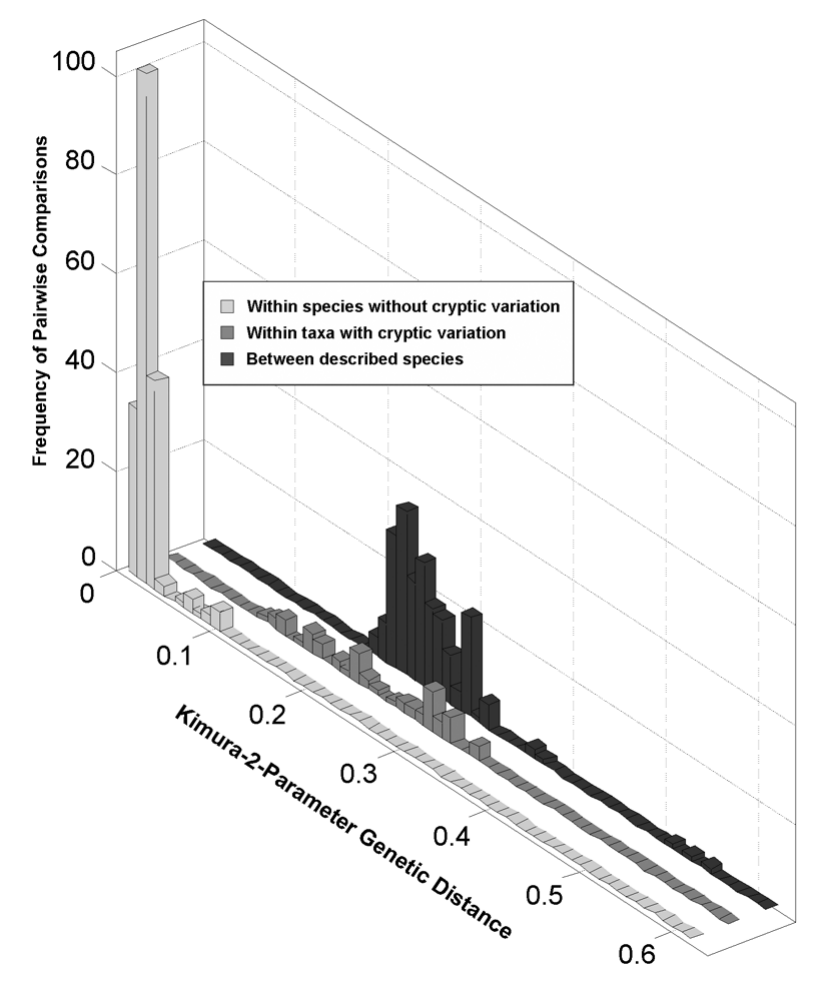
Frequency distributions of pairwise Kimura-2-Parameter (K2P) genetic distances. COI barcode sequences were analyzed to determine frequency distributions of pairwise Kimura-2-Parameter (K2P) genetic distances within and between identified species of halocyprid ostracods analyzed for this study, with separate consideration of taxa (putative species) hypothesized to comprise cryptic species due to large K2P intraspecific distances associated with morphological differences (e.g., presence of different size forms) and/or genetic differentiation between distinct geographic populations (see text for details). Species of *Gigantocypris* (Order Myodocopida) were not included in this analysis.

Kimura-2-Parameter (K2P) genetic distances within species (mean = 0.010 ± 0.017 SD; range 0.000–0.087) were significantly smaller than between species (0.260 + 0.080 SD; range 0.112–1.389; Table 1), excluding eight taxa hypothesized to comprise cryptic species, based on large K2P distances, morphological differences among the specimens examined (especially the presence of different size forms), and/or differences between specimens collected from different geographic regions. K2P genetic distances within and among the cryptic forms of these eight taxa were very similar to those of morphologically-identifiable species, for both within (average = 0.014 + 0.026 SD; range 0.000–0.087) and between species (average = 0.221 ± 0.068; range 0.109–0.329; Table 1). With the removal of these eight taxa, planktonic marine ostracods exhibit a distinct barcode gap (i.e., no overlap between intra- and interspecific genetic distances) [16]as shown in Fig 2.

**Table 1:** Kimura-2-Parameter distances for within and between species comparisons of planktonic ostracods. Intraspecific and interspecific K2P distances were calculated separately for taxa with and without cryptic variation. Eight taxa (putative species) hypothesized to comprise cryptic species-level variation based on markedly high K2P distances were excluded from the first calculation of within and between species variation, which was then calculated for each of the eight taxa individually, with summary statistics for all eight taxa (putative species) hypothesized to comprise cryptic species. Additional information and explanation for many of these taxa is provided in the Results and Conclusions.

	Within Species			Between Species		
	*Avg*	*SD*	*Range*	*Avg*	*SD*	*Range*
**Species with no cryptic variation**	0.010	0.017	0.000–0.087	0.260	0.080	0.112–1.389
**Taxa with cryptic variation**						
*Conchoecia magna*	0.041	0.044	0.000–0.087	0.146	0.049	0.133–0.152
*Deeveyoecia arcuata*	0.003	0.006	0.000–0.012	0.312	0.019	0.295–0.329
*Discoconchoecia elegans*	0.003	0.002	0.000–0.006	0.262	0.038	0.177–0.296
*Halocypris inflata*	0.006	0.004	0.004–0.009	0.090	0.037	0.028–0.118
*Metaconchoecia skogsbergi*	0.004	0.005	0.000–0.011	0.201	0.012	0.189–0.219
*Paraconchoecia oblonga*	0.004	0.004	0.000–0.003	0.318	0.054	0.193–0.197
*Proceroecia brachyaskos*	0.040	0.030	0.006–0.059	0.225	0.027	0.194–0.243
*Proceroecia microprocera*	0.004	0.004	0.000–0.008	0.273	0.007	0.264–0.280
**All taxa with cryptic variation**	0.014	0.026	0.000–0.087	0.221	0.068	0.109–0.329

Despite many barcoding studies of crustaceans [44], few have included marine planktonic ostracods. The patterns and levels of COI barcode variation within and between species determined in this study were consistent with the rather broad range observed for other groups of marine crustaceans [13, 18, 19, 20, 21, 22, 23, 25, 28, 45]. In particular, Blanco-Bercial et al. [23] reported within-species K2P distances for 195 species of planktonic copepods ranging from 0.00–0.15; these authors considered the relatively few K2P values > 0.1 to reflect the presence of cryptic species and/or differentiation among geographic populations. Radulovici et al. [21] reported similar patterns of K2P genetic distances for several crustacean groups, including copepods, both within- (average 0.008) and between-species (average 0.271).

The difficulties of taxonomic identification of living ostracod specimens and the likelihood of complexes of cryptic species in a number of genera add complexity and some uncertainty to our findings. Identified specimens assigned to the same species showing pairwise K2P distance values > 0.1 most frequently showed morphological differences (i.e., the presence of different size forms) between the specimens examined. Genetic divergence between individuals of taxa with different size forms was found in *Discoconchoecia elegans* (average K2P distances between forms = 0.262) and *Metaconchoecia skogsbergi* (0.201), as well as two morphological forms of *Paraconchoecia oblonga* (0.318). Within *Conchoecia magna*, three distinct size classes were distinguished (see S1 Table). K2P genetic distances were notably larger than usual for within-species comparisons between the larger and typical forms (average 0.146), but not between the smaller and typical forms (average 0.030). Between two size forms of *Deeveyoecia (Metaconchoecia) arcuata*, large K2P distances (average 0.312) suggested that in all probability these different size groups are distinct species; the larger form corresponds to the type description, so the smaller form is likely to be a novel species.

In several instances, large within-species distances (K2P > 0.1) were most probably due to genetic differentiation between distinct geographic populations. Examples include *Discoconchoecia elegans* collected from the Arctic and tropical Atlantic (average K2P between collection locations = 0.262) and several species collected from both the Sargasso Sea and Eastern Atlantic, including *Deeveyoecia (Metaconchoecia) arcuata* (0.312); *Proceroecia brachyaskos* (0.225); *Proceroecia microprocera* (0.273); and *Porroecia spinostris* (0.074). However, some species showed little or no genetic divergence among specimens from difference ocean regions, including: *Alacia valdiviae* collected from the NE Atlantic and Southern Ocean (0.002) and *Halocypria globosa* from the SE Atlantic and South Indian Ocean (0.009).

### Neighbor Joining gene tree

**Fig 3:**
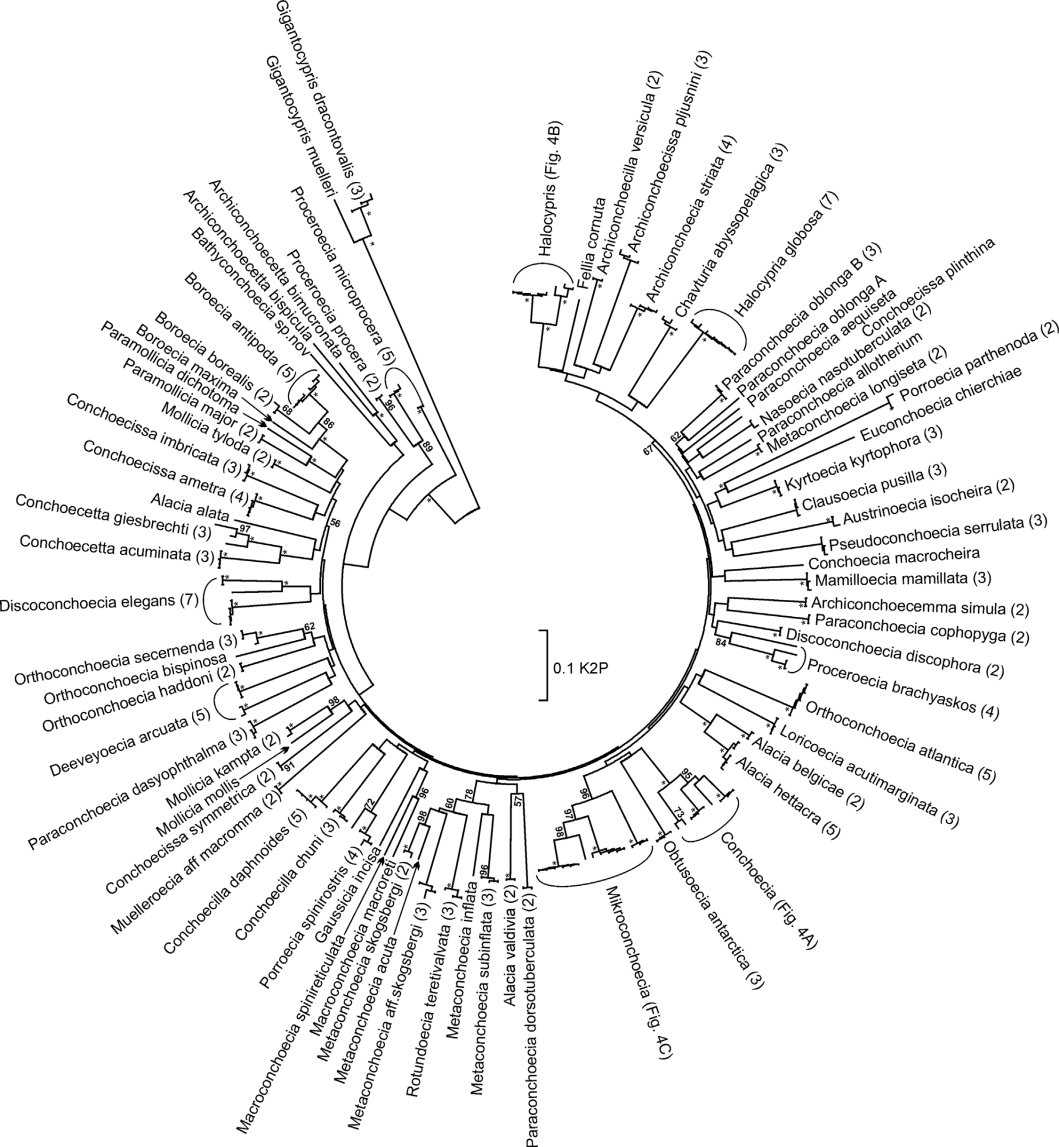
COI gene tree for specimens of planktonic marine ostracods analyzed in this study. Neighbor Joining gene tree based on COI barcode region sequences for 210 specimens of 78 species of ostracods. Analysis used Kimura-2-Parameter distances; bootstrap values > 50% for 1,000 sub-replicates are displayed at nodes; bootstrap values = 99% are indicated by an asterisk (*).

Nearly all ostracod species were resolved as groups based on analysis of COI barcode sequence variation; the Neighbor Joining (NJ) gene tree shows high bootstrap support (usually 99%) for described species (Fig 3). In contrast, only a few genera were resolved, usually with low bootstrap support on the NJ gene tree. The most notable exception is *Gigantocypris* (mean K2P between all other species = 0.55 ± 0.06 SD), which belongs to a different order of pelagic ostracods (Order Myodocopida) than that to which all other genera analyzed belong (Order Halocyprida).

### Integrative taxonomic analysis for selected genera

**Fig 4:**
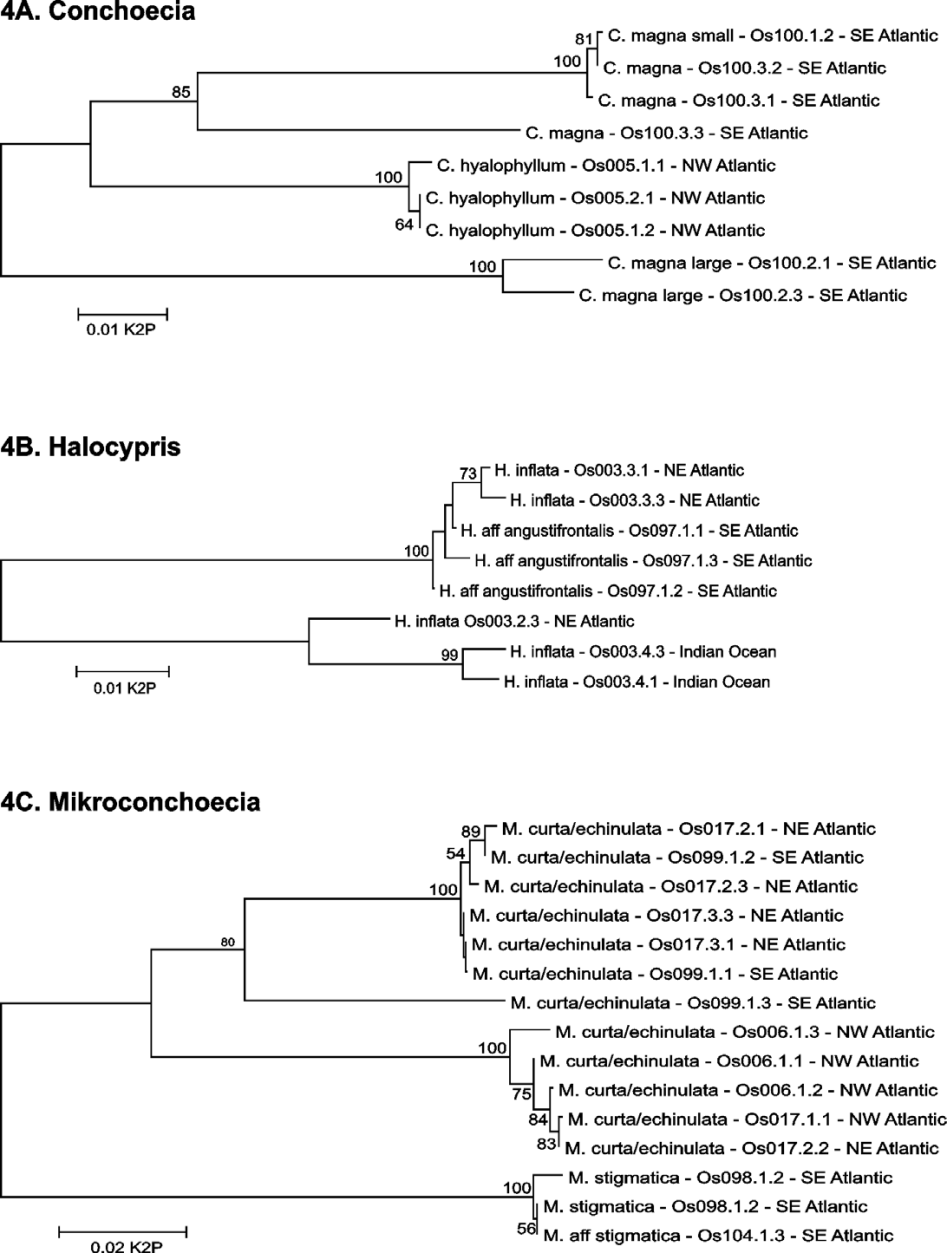
COI gene trees for selected genera. Detailed views of the Neighbor Joining tree are shown for the following genera: A) *Conchoecia*, B) *Halocypris*, and C) *Mikroconchoecia*. Trees were analyzed and shown as explained in the legend to Fig 3.

Several ostracod genera exhibit systematic complexities or uncertainties that continue to provide challenges for taxonomic analysis, including interpretation of results from integrative morphological–molecular approaches. These are described here in more detail:

- *Boroecia*: This genus exemplifies some of the prevailing taxonomic uncertainties in halocyprids. The first of species in the genus to be described was *B*. (*Conchoecia*) *borealis* (Sars, 1866), which is one of the dominant species in the mesopelagic assemblage in the Norwegian Sea and sub-boreal North Atlantic [46]. Subsequently two further species were described as *B*. *maxima* (Brady and Norman, 1896) and *B*. *antipoda* (Müller, 1906). The former species was originally described based on specimens from two localities in the Greenland Sea (74°N) and Faeroe Channel (~60°N), but no type specimen was designated. It is now known to be the most abundant halocyprid species in the high Arctic [47]. The latter species is a Southern Ocean endemic [11], but has erroneously been identified from the tropical Pacific [48]. For several decades these two species were considered to be subspecies of *B*. *borealis;* a confusion that has only recently been fully resolved [49]. A smaller form of *B*. *maxima* has been identified (but not formally described) from the north of the Bering Strait, and two further species await formal description–one from the North Pacific (Gulf of Alaska) together with the specimens erroneously attributed to *B*. *antipoda* [48]. COI sequences are reported for the three described species here (Fig 3, S1 Table), although analysis of additional specimens to resolve remaining taxonomic uncertainties is needed for determination of a definitive barcode sequence for *B*. *maxima*.
- *Conchoecia*: Angel [50] provided a full description of *Conchoecia magna*, the type species for the genus. Both large and small forms of the species have subsequently been noted. COI barcode sequence variation clearly resolves two groups; large specimens are grouped together and are distinct from the typical form (average K2P distance = 0.146); the typical and small forms do not differ genetically (Table 1, Fig 4A).
- *Discoconchoecia*: Species in this genus were originally included in *Paraconchoecia* [48] and were split off by Martens [51]. It includes one of the first halocyprid species to be described, *Discoconchoecia elegans* (Sars, 1866). This species has been reported from all oceans, mostly from high latitudes, but also from mesopelagic zones in tropical waters. Two different size forms were collected from different geographic locations: a small form (females 1.20–1.34 mm; males 1.12–1.30 mm) from low latitudes (< 30°N) in the Atlantic, while a larger form (females 1.48–1.94 mm; males 1.68–1.96 mm) consistent with the original description of the species from the vicinity of the Lofoten Islands, Norway [52] was collected from high latitudes (> 50°N). The size forms also exhibit minor morphological differences in carapace length/height ratios, spination at the posterior dorsal corner of the carapace, and morphologies of the frontal organs. However, these are yet to be formally described. COI barcode variation discriminated the mid-latitude Atlantic form from those of the Gulf of Alaska (K2P average 0.263) and the Greenland Sea (K2P average 0.259). The large genetic distances and the NJ tree topology (Fig 3) suggest the presence of a number of geographically distinct, cryptic species within *D*. *elegans* [53].
- *Halocypris*: Species of *Halocypris* are very difficult to distinguish morphologically. Two species have been reported to be abundant in the epipelagic and shallow mesopelagic of the subtropical Atlantic, *H*. *pelagica* and *H*. *inflata* [5]. The species lack diagnostic characters in their external morphology, but can be differentiated on the basis of carapace size. There is a degree of geographical and bathymetric separation between the species; where they do co-occur there is evidence of carapace size displacement [54]. Chavtur and Stovbun [55] described an additional species in the Pacific Ocean, *H*. *angustifrontalis*, which is also morphologically very similar, though slightly larger in size than its congeners. Two *Halocypris* species were analyzed in this study (see S1 Table). Larger specimens from a deep sample (4,000–5,000 m) collected in the South Atlantic were provisionally identified as *H*. aff. *angustifrontialis*. Specimens identified as *H*. *inflata* yielded COI sequences that were resolved into three distinct clades: one comprised of specimens collected from the Southern Ocean, and two of Atlantic samples, one of which one was closely aligned with *H*. aff. *angustifrontialis*. Both the NJ tree topology (Fig 4B) and large K2P distances between forms (average 0.090) suggest the presence of cryptic species within *H*. *inflata*. Based on COI barcode variation, there may be a number of species in this complex, some of which are undoubtedly novel. Clearly, a number of taxonomic challenges remain and additional morphological and molecular analysis is required.
- *Metaconchoecia* and allied genera: The genus *Metaconchoecia* continues to present many taxonomic challenges; recent re-examination resulted in subdivision into eight new genera based on morphological criteria [56]. A number of species continue to be taxonomically questionable. Among eight species previously assigned to this genus analyzed in this study, several could be identified with confidence: *Clausoecia pusilla*, *Kyrtoecia kyrtophora*, *Nasoecia nasotuberculata*, *Rotundoecia teretivalvata*, and *Metaconchoecia subinflata* (S1 Table). Questions of the taxonomic significance of carapace size for these taxa prevent designation of definitive barcode sequences in a number of instances. Three distinct size forms of *M*. *skogsbergi* have been reported with slightly different bathymetric and zoogeographical ranges; these will likely prove to be different species, but have not yet been formally recognized. In addition the original species description of *M*. *skogsbergi* was based on a secondary source with no type locality or material being defined, hence which of these three forms is typical is uncertain. Two size forms of *Deeveyoecia (Metaconchoecia) arcuata* have been recognized and likely represent distinct species (see above).
- *Mikroconchoecia*: Of three species analyzed in this study, only one species, *M*. *stigmatica*, could be identified unambiguously without dissection, and hence is the only species which could be confidently identified alive. Two more common shallow mesopelagic species, *M*. *curta* and *M*. *echinulata*, could not be distinguished. These two species are very small (< 0.9mm), extremely similar morphologically, display only slight differences in size, and have overlapping geographical and bathymetric ranges. COI barcode analysis of specimens identified as *M*. *curta/echinulata* indicated that two species may in fact be present (K2P distances up to 0.146; Fig 4C), but the failure to accurately identify the specimens prevented the assignment of definitive barcode sequences to these two species.
- *Paraconchoecia*: This halocyprid genus is in need of taxonomic revision and many species are expected to require reclassification; they all show clear morphological differences from *P*. *spinifera*, the type species. This genus was established by Claus [57] and the species were subsequently combined within a single large halocyprid genus, *Conchoecia* (Műller 1906). Poulsen [48] re-instated the genus, including 22 species, of which many have since been split off into other genera, including *Discoconchoecia*, *Porroecia*, *Proceroecia*, and *Mamilloecia*. Among seven species analyzed in our study, three species, *P*. *cophopyga*, *P*. *dasyophthalma*, and *P*. *dorsotuberculata*, had typical within-species K2P distances (average 0.006). Two forms of *Paraconchoecia (Conchoecia) oblonga* were distinguished on the basis of a single, but very obvious, external morphological character [58]; detailed examination is revealing that there are additional morphological differences. The typical ‘form A’ is more abundant in the Eastern Atlantic, whereas ‘form B’ predominates in the Western Atlantic. Where they co-occur, COI sequence divergence (average 0.138) suggests that the two forms are separate species. In sum, the current concept of this genus is highly polyphyletic and is in need of significant revision.

### MOTUs versus described species

**Fig 5:**
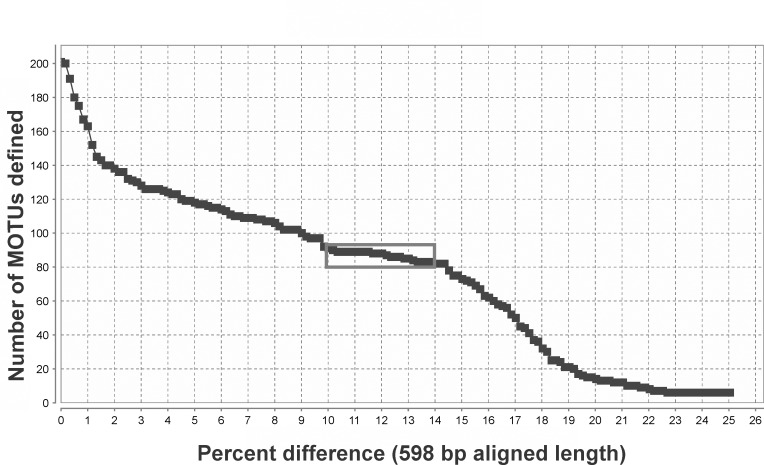
Comparison between numbers of MOTUs and described species. Number of MOTUs inferred from the barcode alignment trimmed to 598 base-pairs using a range of cut-offs (x-axis) expressed as percentage differences. The analyzed data include 78 described species, of which eight are hypothesized to include two or three cryptic species. The box defines a flat section of the graph ranging from 10% sequence difference (defining 91 species) to 14% (82 species).

Results of the jMOTU [43] analysis show an attenuation of the slope indicating a within-species MOTU threshold of 1.0–1.5% (Fig 5). A flat section of the curve bounded by 10% and 14% sequence differences is consistent with a 13% sequence difference threshold level for species differentiation [13]. This range of MOTU discrimination levels corresponds to numbers of MOTUs defined, as follows: 10% sequence difference (defining 91 species); 11% (89); 12% (88); 13% (85); and 14% (82). Such analysis provides support for the existence of some–but not a large number–of genetically-distinct forms or cryptic species.

### Remaining challenges for integrativetaxonomic analysis of ostracods

Morphological species identification of ostracods will continue to present challenges for 'gold standard' barcoding studies [59] that seek to match DNA sequences to specimens that have been accurately identified based on morphological characters. Importantly, morphologically-distinct planktonic marine ostracod species–even closely-related species–were resolved into distinct clades in tree-based analyses with high levels of confidence (Fig 3). This finding provides confidence that unknown specimens can be identified based on the COI barcode sequence, although if, and only if, that species is present in the barcode database [23]. However, as is usual for the COI barcoding region, relationships among genera and higher groups were not resolved (Fig 3), so specimens of species for which no barcode sequence is known can neither be identified nor classified based on COI variation. New insights into the deeper phylogenetic relationships of the ostracoda as a group, including resolution of genus-level and above, will require multi-gene or phylogenomic approaches.

Inevitably for such a poorly-understood taxonomic group as marine planktonic ostracods, there are significant challenges, impediments, and limitations on new advances in understanding based on integrative morphological-molecular taxonomic analysis. Exceptionally among ecologically-important marine zooplankton groups, ostracods have not been well studied, resulting in an inadequate foundation for definitive taxonomic and systematic analysis. Several ostracod genera are clearly polyphyletic; many species have been inadequately described; closely-related species cannot readily be discriminated based on the available diagnostic characters [1,6].

A significant impediment to progress in the use of DNA barcoding for taxonomic analysis of ostracods is that accurate identification of living specimens is required, which has to be based on external morphological characters, and the discrimination of several taxa requires dissection and careful microscopic examination. In the future, techniques to remove DNA without damaging diagnostic morphological characters, such as those developed for copepods [60], which allow subsequent morphological taxonomic identification, may prove possible for ostracods. For this study, the need for examination of living species resulted in the small numbers of individuals analyzed for some species, which is not ideal for statistical analysis and may result in errors and misinterpretation of results. However, given the paucity of available barcode data for this systematically complex and challenging group, these data provide a useful foundation for subsequent integrative morphological–molecular taxonomic studies.

The range of levels and patterns of genetic divergence among marine planktonic ostracods reflect the dynamic evolutionary processes that impact species in every environment, including the pelagic [61]. Comprehensive analysis of the COI barcode region for marine planktonic ostracods may thus be expected to resolve some–but not all–of the challenges in meeting the goal of a comprehensive understanding of species diversity and distribution, based upon accurate and reliable identification and discrimination of species of this ubiquitous, abundant, and ecologically-important group of marine zooplankton.

## Conclusions

DNA sequences of the cytochrome oxidase subunit I (COI) barcode region are reported for 210 specimens of 78 species of marine planktonic ostracods, including two novel species, and 51 species for which barcodes have not been previously published. COI barcodes showed significantly different, non-overlapping differences within- versus between-species for all species for which specimens could be confidently identified. Eight putative species hypothesized to comprise cryptic species, based on morphological variation (size forms) and/or differentiation of geographic populations, were analyzed separately and showed similar patterns and levels of genetic distances. Despite lack of accurate species descriptions, absence of diagnostic morphological characters for species identification, and very incomplete knowledge of generic boundaries and systematic relationships, integrative morphological–molecular taxonomic approaches will allow routine analysis and can yield new understanding of the diversity, distribution, and ecological importance of ostracods in the marine planktonic assemblage. A Neighbor Joining COI gene tree resolved nearly all described species analyzed, with multiple sequences forming clusters with high bootstrap values. Based on taxonomically and geographically extensive sampling and analysis (albeit with small sample sizes), the COI barcode region was shown to be a valuable character for discrimination, recognition, identification, and discovery of species of marine planktonic ostracods.

## Supporting Information

S1 Table Collection information and metadata for specimens of marine planktonic ostracods analyzed for this study. All specimens analyzed are listed by species name, with DNA voucher numbers, GenBank Accession Numbers, and collection information (date, latitude and longitude, ocean region and cruise). Additional metadata are included in each GenBank entry.
(DOC)
