# Supplementary material for: Identification, Discrimination, and Discovery of Species of Marine Planktonic Ostracods Using DNA Barcodes
Source: PLoS One. 2016 Jan 5;11(1):e0146327. doi: 10.1371/journal.pone.0146327 (PMC4701487; doi:10.1371/journal.pone.0146327)
Supplement: S1 Table — All specimens analyzed are listed by species name, with DNA voucher numbers, GenBank Accession Numbers, and collection information (date, latitude and longitude, ocean region and cruise). Additional metadata are included in each GenBank entry. (DOC) [file pone.0146327.s001.doc]

**Supporting Information**

**S1 Table. Collection information and metadata for specimens of marine planktonic ostracods analyzed for this study**. All specimens analyzed are listed by species name, with DNA voucher numbers, GenBank Accession Numbers, and collection information (date, latitude and longitude, ocean region and cruise). Cruise numbers are explained in the text. Additional metadata are included in each GenBank entry.

Footnote: Asterisks (*) indicate previously published GenBank Accession Numbers [28].

**S1 Table (continued)**

**S1 Table (continued)**

**S1 Table (continued)**

**S1 Table (continued)**
